# Whole genome sequence and manual annotation of *Clostridium autoethanogenum*, an industrially relevant bacterium

**DOI:** 10.1186/s12864-015-2287-5

**Published:** 2015-12-21

**Authors:** Christopher M. Humphreys, Samantha McLean, Sarah Schatschneider, Thomas Millat, Anne M. Henstra, Florence J. Annan, Ronja Breitkopf, Bart Pander, Pawel Piatek, Peter Rowe, Alexander T. Wichlacz, Craig Woods, Rupert Norman, Jochen Blom, Alexander Goesman, Charlie Hodgman, David Barrett, Neil R. Thomas, Klaus Winzer, Nigel P. Minton

**Affiliations:** BBSRC/EPSRC Synthetic Biology Research Centre, School of Life Sciences, University of Nottingham, Nottingham, NG7 2RD UK; School of Pharmacy, University of Nottingham, Nottingham, NG7 2RD UK; School of Chemistry, University of Nottingham, Nottingham, NG7 2RD UK; Bioinformatics and Systems Biology, Justus-Liebig-University Giessen, 35392 Giessen, Germany

**Keywords:** *Clostridium autoethanogenum*, Next generation sequencing, Acetogen, Manual annotation, Synthesis gas fermentation

## Abstract

**Background:**

*Clostridium autoethanogenum* is an acetogenic bacterium capable of producing high value commodity chemicals and biofuels from the C1 gases present in synthesis gas. This common industrial waste gas can act as the sole energy and carbon source for the bacterium that converts the low value gaseous components into cellular building blocks and industrially relevant products via the action of the reductive acetyl-CoA (Wood-Ljungdahl) pathway. Current research efforts are focused on the enhancement and extension of product formation in this organism via synthetic biology approaches. However, crucial to metabolic modelling and directed pathway engineering is a reliable and comprehensively annotated genome sequence.

**Results:**

We performed next generation sequencing using Illumina MiSeq technology on the DSM10061 strain of *Clostridium autoethanogenum* and observed 243 single nucleotide discrepancies when compared to the published finished sequence (NCBI: GCA_000484505.1), with 59.1 % present in coding regions. These variations were confirmed by Sanger sequencing and subsequent analysis suggested that the discrepancies were sequencing errors in the published genome not true single nucleotide polymorphisms. This was corroborated by the observation that over 90 % occurred within homopolymer regions of greater than 4 nucleotides in length. It was also observed that many genes containing these sequencing errors were annotated in the published closed genome as encoding proteins containing frameshift mutations (18 instances) or were annotated despite the coding frame containing stop codons, which if genuine, would severely hinder the organism’s ability to survive. Furthermore, we have completed a comprehensive manual curation to reduce errors in the annotation that occur through serial use of automated annotation pipelines in related species. As a result, different functions were assigned to gene products or previous functional annotations rejected because of missing evidence in various occasions.

**Conclusions:**

We present a revised manually curated full genome sequence for *Clostridium autoethanogenum* DSM10061, which provides reliable information for genome-scale models that rely heavily on the accuracy of annotation, and represents an important step towards the manipulation and metabolic modelling of this industrially relevant acetogen.

**Electronic supplementary material:**

The online version of this article (doi:10.1186/s12864-015-2287-5) contains supplementary material, which is available to authorized users.

## Background

One of the greatest challenges facing industry and society is the future sustainable production of chemicals and fuels from non-food resources while at the same time reducing greenhouse gas emissions. To date, the focus has been on the use of lignocellulosic biomass feedstocks. The exploitation of biomass, however, is reliant on an energy intensive pre-treatment step, and thereafter, the addition of costly exogenous hydrolytic enzymes required to convert the partially deconstructed biomass into the sugars needed by the fermentative process organisms. The costs involved are making the development of economic processes extremely challenging [1, 2]. A range of solutions are being explored to increase the economic viability of this process, including the direct microbial conversion of biomass by lignocellulose degrading organisms [3]. One alternative solution is to develop processes based on acetogenic bacteria such as *Clostridium autoethanogenum*, whereby carbon is directly captured (in the form of carbon monoxide or carbon dioxide) through anaerobic gas fermentation. These bacteria are capable of growth on a spectrum of waste gases from industry (e.g. steel manufacture and oil refining, coal and natural gas [4–7]). Thus, gas fermentation allows the production of low carbon fuels and high-value chemicals without competing for food or land. It therefore represents an extremely versatile platform for the sustainable production of commodity chemicals and fuels.

*C. autoethanogenum* is a strictly anaerobic, Gram-positive, spore forming, rod-like, motile bacterium. It was first isolated from rabbit faeces in 1994 under an atmosphere of carbon monoxide, nitrogen and carbon dioxide, with carbon monoxide as the sole energy source [8, 9] and was identified as a facultative chemolithotroph [9]. Since its isolation, this bacterium has quickly gathered interest as a potential chassis for biofuel and high-value chemical production (see for example [7, 10–13]).

As a means of further understanding this organism, and for its effective exploitation for biofuel and biochemical production by means of metabolic engineering, a draft genome sequence of *C. autoethanogenum* DSM10061 was first elucidated using 454 GS FLX Titanium and Ion Torrent PMG techniques by Bruno-Barcena et al. in 2013 [14]. The collection of contigs is available under the NCBI accession number GCA_000427255.1. Subsequently, Pacific Biosciences single-molecule DNA sequencing technology [15] was used to generate a finished genome sequence by Brown et al. (2014) that is accessible under NCBI accession number GCA_000484505.1 [16]. According to this sequence, the bacterium has a chromosome length of 4,352,205 base pairs, with 4161 predicted genes, 4042 of which are potentially protein-coding genes with 18 pseudogenes present, and 18 RNA genes. Raw data from a range of sequencing techniques used by the same group has recently been published, and includes Roche 434, Illumina Truseq technology, Ion torrent, PacBio RS II, and Sanger sequencing datasets [17], however the deposited sequence is presently exclusively representative of the PacBio sequencing data.

In recent years, the field of next-generation sequencing has become more accessible and technologies continue to evolve at a dramatic pace, and as such many previously published genomes which have been revisited, have been updated and improved [18–20]. Improvements in both sequencing technologies and analysis tools have enabled a higher confidence in the generated genome sequence, and as such the coding sequence annotations also become more accurate and refined. Revisiting and updating existing genome annotations is absolutely essential, as not only does it allow the opportunity to increase the users understanding of the organism in question, but it also improves accuracy for downstream users when performing automated annotations of related species [21, 22], reducing the introduction of errors caused by historic sequencing inaccuracies. Revisiting existing annotations also allows application of new biological knowledge to previously uncharacterised loci, and in the case of manual annotation, allows the opportunity to standardise features such as enzymes names and functional characterisation, for better integration with models.

Our detailed inspection of the Brown et al. closed genome sequence identified multiple instances of coding DNA sequences that were annotated as containing frame-shift mutations, where the reading frame had become disrupted. Additionally, the coding region of many genes appeared to contain premature stop-codons when compared to those of the closely related acetogenic species *Clostridium ljungdahlii,* accessible under NCBI accession number GCA_000143685.1 [23], thus theoretically truncating the protein products. Were these frame-shifts genuine it would have the effect of severely debilitating the organism’s capacity to survive. This includes the ATP–dependent DNA helicase RecQ, an important protein in genome maintenance, which appeared to contain a stop codon which truncated the full length protein into two 280 and 433 amino acid products (CAETHG_0594 and CAETHG_0595). To further understand these apparent frame-shifts, we sequenced a stock of *C. autoethanogenum* DSM10061, purchased directly from the DSMZ culture collection, using Illumina MiSeq technology and mapped these reads onto the Brown et al*.* finished genome sequence. We found 243 discrepancies compared to the finished genome sequence, the vast majority of which had the effect of ‘repairing’ the annotated frame-shift regions and premature stop-codons identified in the genome. Importantly, many of those genes exhibit important cellular functions including the C1 metabolism underlying gas fermentation.

In the following sections, we focus on the identified differences, resulting in altered or newly introduced functional annotations and their consequences for the protein network in *C. autoethanogenum*, and on the underlying reasons for these discrepancies. We also highlight a possible shortcoming of the PacBio RS II sequencing technology, which has implications for future users wishing to employ this technology for gap closing when performing de-novo sequencing. We demonstrate the importance of employing a further sequencing technology following gap closure by PacBio RS II in order to generate a sequence with a high confidence level, and in doing so we have corrected 142 annotation errors in protein coding sequences brought about through apparent frameshift mutation due to under-called homopolymer regions. In culmination of our analysis, we present a corrected and fully manually curated genome for *C. autoethanogenum*, a step which enables a downstream user to have confidence in the annotation, as a purely automated annotation can often propagate previous errors made during annotation of related species [24], and which allows the annotation to be presented in a uniform and standardised manner. This represents an important step towards accurate manipulation of the industrially relevant organism, and which may be reliably used as a basis for the generation of metabolic and genomic models.

## Results

### Analysis of the *C. autoethanogenum* genome by Illumina sequencing reveals 243 discrepancies from the Brown et al. finished genome sequence

**Fig. 1:**
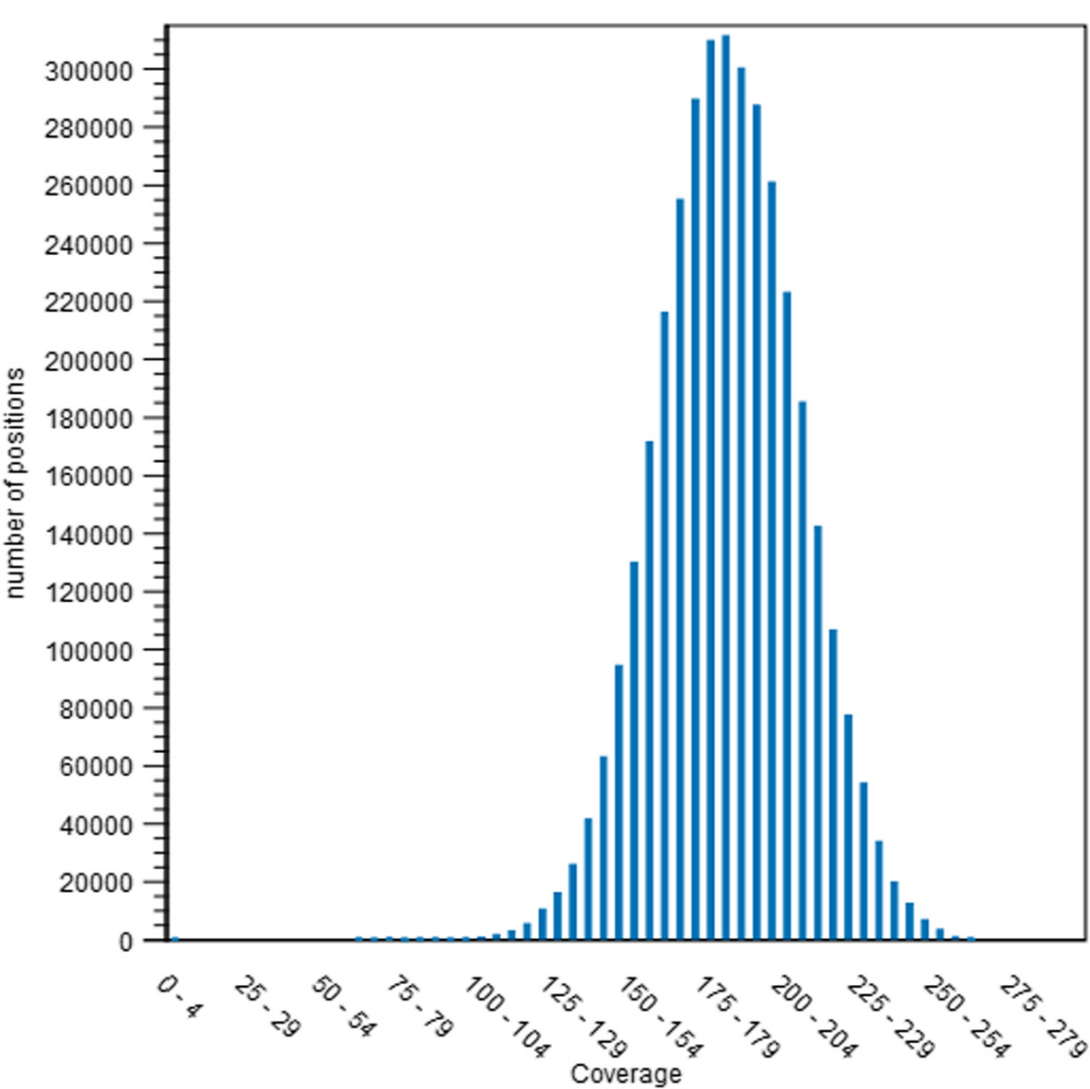
Distribution of coverage of coding sequences across the genome. A visual representation of the depth of coverage of all coding sequences as generated by the Brown et al. genome annotation

**Fig. 2:**
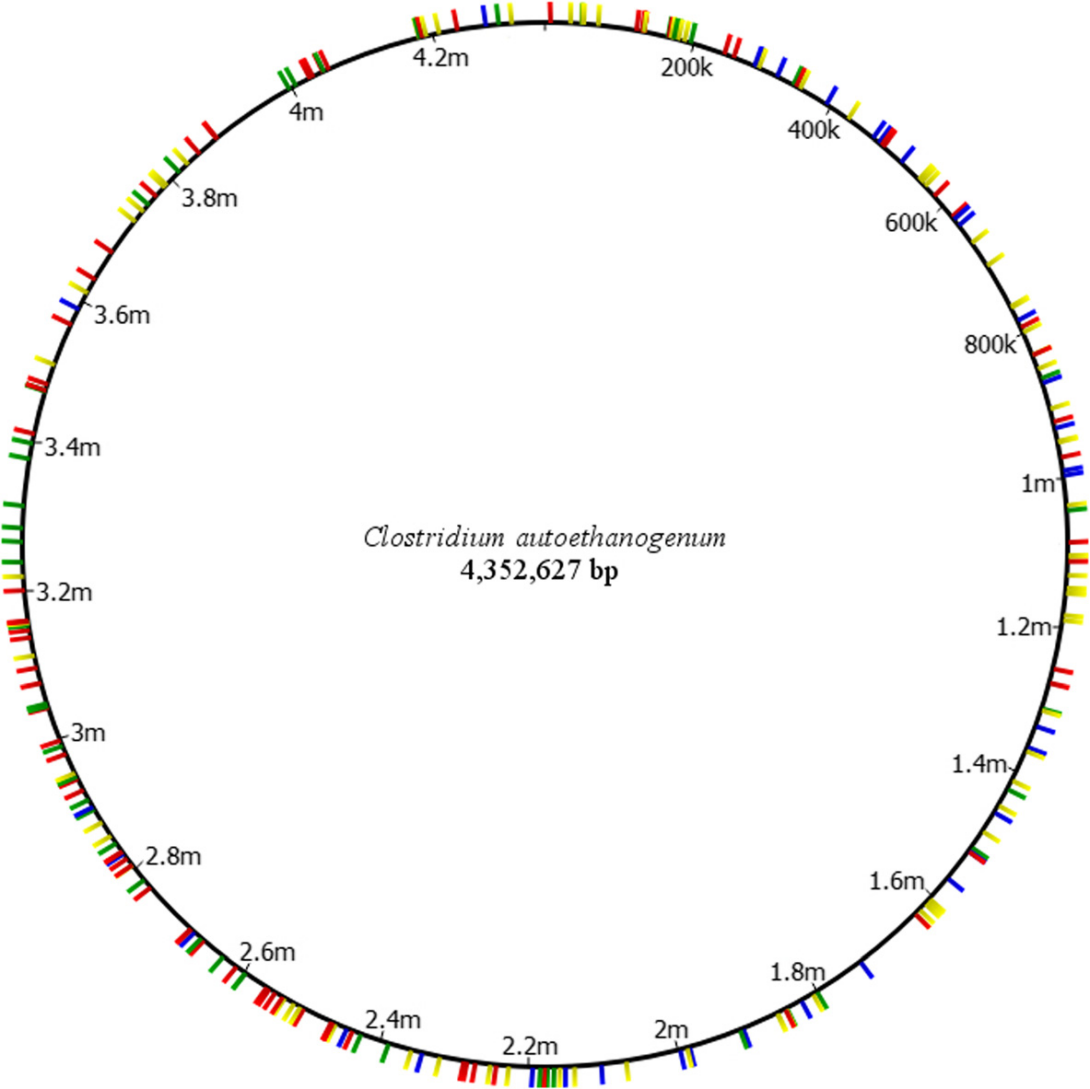
Locations of the 243 insertion sites across the genome. Highlighted areas display the location of an insertion site as detected by our Illumina resequencing of the DSM10061 strain when compared to the Brown et al*.* sequence

Following our initial observations of a number of frameshift annotations of the published genome for *C. autoethanogenum*, and to confirm that our stock of the organism was representative of the published strain, we performed Illumina MiSeq on our DSM10061 strain acquired directly from the DSMZ. This generated over 3.5 million mapped short reads with an average length of 249.91 base pairs, resulting in an average coverage of 200.96 with a standard deviation of 25.67. To analyse the range of the coverage across the genome, we assessed coverage of specifically the coding regions present in the Brown et al. finished genome sequence. The results confirmed that 99.85 % of CDS’s had coverage of at least 40 reads for 100 % of the sequence, and 100 % of CDS’s had coverage of at least 40 reads for at least 60 % of the sequence. The distribution of coverage against all coding regions is represented graphically below (Fig. 1.). It was found previously that de-novo assembly using an Illumina MiSeq dataset led to regions of low coverage [16], however using the PacBio generated sequence as a reference appears to have alleviated this problem. The genome had 4,352,627 base pairs, a G + C content of 31.09 %, predicted 3969 protein-coding sequences (CDS), and 70 RNA-coding genes. The whole-genome sequencing project for *C. autoethanogenum* JA1-1 has been deposited at National Center for Biotechnology Information (NCBI) under the accession number CP012395. We were able to map reads using the Brown et al. finished genome sequence as the reference and the software tool CLC Genomics Workbench version 7.0 (CLC Bio; Qiagen). We observed 243 differences in our strain as compared to the reference (Table 1, Additional file 1). Interestingly, all but one of these discrepancies were identified as single base pair insertions, and these occurred with a seemingly random distribution across the genome, both in coding and non-coding regions (Fig. 2). Large INDELs were also screened for using the CLC genomics workbench, but none were detected.

**Table 1:** Comparison of the discrepancies occurring between the current and Brown et al*.* whole genome sequencing of *C. autoethanogenum*

Position	Insertion	Gene	Homopolymer length		Amino acid length		Sequence identity		
			CLAU	CLJU	CLAU	BRO	BRO	CAUT	CLJU
46129	T	CAETHG_0051	6	6	412	412	119/367	NF	412/412
283331	C	CAETHG_0263	5	5	370	370^a^	NF	370/370	369/370
627984	C	CAETHG_0567	2	2	521	245	231/233	NF	521/521
656810	T	CAETHG_0595	6	6	722	279	269/269	722/722	717/722
928129	C	CAETHG_0862	5	5	293	250	249/249	NF	293/293
985484	C	CAETHG_0915	4	4	688	688	NF	NF	688/688
1106176	A	CAETHG_1030	6	6	172	126	109/109	NF	172/172
1457002	C	CAETHG_1363	6	6	296	254	249/249	294/295	292/296
1603900	T	CAETHG_1501	8	8	401	401	NF	NF	401/401
1620246	T	CAETHG_1521	6	NF	323	316	315/315	323/323	310/323
2222019	T	CAETHG_2078	8	8	445	326	325/325	NF	444/445
2352969	T	CAETHG_2212, CAETHG_2213	2	2	416	202	None	416/416	414/416
2596835	G	CAETHG_2429	7	7	400	382	378/378	400/400	400/400
2683087	C	CAETHG_2503	4	4	640	615	601/605	640/640	639/640
2805023	A	CAETHG_2601, CAETHG_2602	7	AAAGAAA	370	141	138/138	370/370	328/366
2852812	T	CAETHG_2647	8	NF	470	314	314/314	469/470	NF
3076804	A	CAETHG_2840	8	8	635	487	482/483	635/635	635/635
3396986	G	CAETHG_3132, CAETHG_3133	5	5	160	152	149/149	160/160	160/160
3468796	G	CAETHG_3212	5	5	271	291	270/271	270/271	270/271
3752592	G	CAETHG_3500	5	5	459	418	413/415	459/459	459/459
3786709	T	CAETHG_3531	6	NF	144	64	64/64	144/144	NF
3877937	A	CAETHG_3599	3	3	270	74	181/182	270/270	269/270
3994749	G	CAETHG_3707	6	6	261	176	172/177	NF	261/261
4180142	T	CAETHG_3902	5	5	359	99	94/95	NF	359/359

This table shows a representation of the discrepancies that occur when the current Illumina sequence (CLAU) is mapped against the published Brown et al. sequence (BRO). The insertion column describes the mutation occurring in the CLAU genome compared to the BRO genome. Homopolymer length indicates the number of the same base occurring consecutively at the site of the discrepancy. Amino acid length gives the annotated protein length of the gene in which the discrepancy occurs. The sequence identity is relative to our *C. autoethanogenum* genome sequence when protein BLAST searched on the NCBI database. CLAU, *C. autoethanogenum* finished genome sequence in present study; CLJU, *C. ljungdahlii* DSM 13528 finished genome sequence (GCA_000143685.1); BRO, Brown et al*. C. autoethanogenum* finished genome sequence (GCA_000484505.1); CAUT, Bruno-Barcena et al. *C. autoethanogenum* draft genome sequence (GCA_000427255.1); *NF* not found. ^a^indicates protein codes for multiple stop codons

**Table 2:** A summary of the CLAU genome characteristics following manual annotation

Attribute	Genome (total)	
	Value	% of total
Size (bp)	4352627	N/A
G + C content (bp)	1353310	31.09
Coding region (bp)	3686220	84.69
Total genes	4039	N/A
RNA genes	70	17.33
Genes with GO number(s)	2331	57.71
Genes with SignalP hits	194	4.80
Genes assigned to COGs	36	0.89
CDS with 0 conserved domains	866	21.82
CDS with 1 conserved domains	1983	49.96
CDS with 2 conserved domains	810	20.41
CDS with 3 conserved domains	211	5.32
CDS with 4 conserved domains	62	1.56
CDS with more than 4 conserved domains	37	0.93
Genes with signal peptides	194	4.80
Genes with transmembrane helices	1074	26.59

**Fig. 3:**
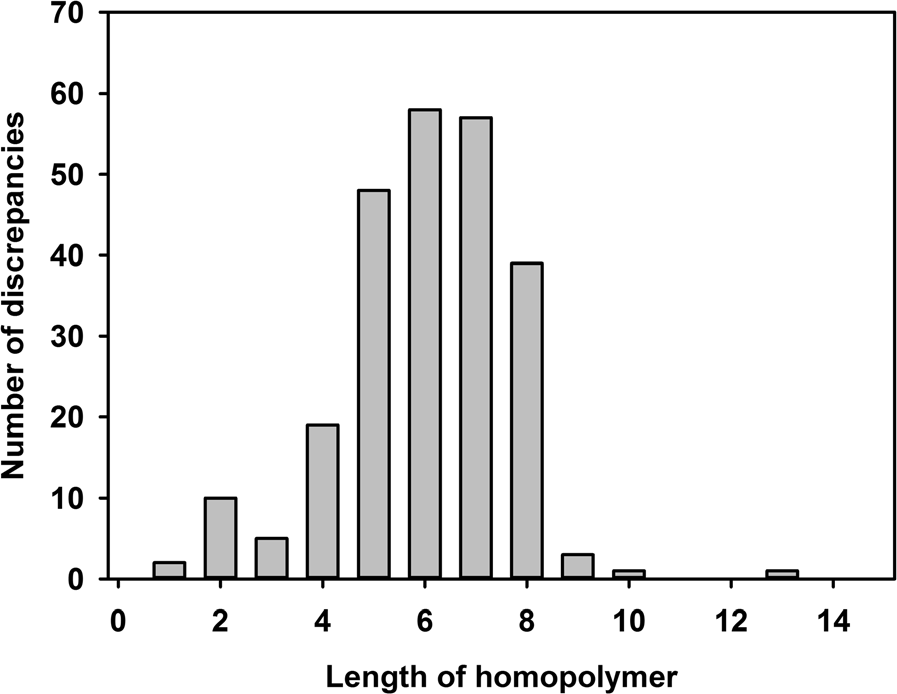
Discrepancies as related to homopolymer length. The length of the homopolymer where each discrepancy was determined and data collated. The vast majority of discrepancies were found to occur when homopolymer length was between 4 and 8

### Sanger sequencing confirms single base pair insertions

To determine whether these discrepancies were genuine differences or artefacts of the sequencing technology employed, we performed further analysis on a randomly selected sample, from those which occurred in coding regions, by Sanger sequencing. Primers were designed approximately 250 base pairs upstream and downstream of the site in question, and the resultant ~500 base pair amplified product was sequenced using both forward and reverse primers by Source Bioscience. Sanger sequencing from both forward and reverse reactions from all samples confirmed our Illumina MiSeq data (Additional file 2), indicating that the Illumina sequencing had made the correct calls for these single base discrepancies in our strain versus the Brown et al. finished genome sequence.

### Comparison of our sequence to that of published sequences revealed that a high percentage of our discrepancies agreed with the finished genome sequence of *C. ljungdahlii* and of genes located in the Bruno-Barcena draft genome sequence of *C. autoethanogenum*

The finished whole genome sequence (WGS) of *C. ljungdahlii,* a genus of *Clostridium* that is phylogenetically indistinguishable from *C. autoethanogenum* [25] and contains a very high genome sequence similarity (>98 %, [26, 27]), was published in 2010 by Köpke et al*.*, and is available on the NCBI database (NCBI: NC_014328.1 [5]). We used the nucleotide Basic Local Alignment Search Tool (BLAST) to search for homologous regions in *C. ljungdahlii* to those containing discrepancies between our finished genome sequence and the Brown et al*.* finished genome sequence of *C. autoethanogenum* to determine which the corresponding sequences from *C. ljungdahlii* that are present agree with. We found that of the 225 regions that are present in *C. ljungdahlii* all instances confirm our *C. autoethanogenum* finished genome sequence. Furthermore, we performed protein BLAST searches against the amino acid sequences of each discrepancy that occurred within a protein-coding region (142 in all). Of these, 127 coding regions are also present in *C. ljungdahlii,* and 59 are represented within the contigs of the Bruno-Barcena draft genome sequence. 125 coding sequences from *C. ljungdahlii* are identical to those found in our finished genome sequence, and the two that were not also did not agree with the Brown et al. finished genome sequence. Of the 59 coding-regions present within contigs of the Bruno-Barcena draft genome sequence, 55 agreed with our sequencing and the four that did not also did not agree with the Brown et al*.* finished genome sequence (Table 1, Additional file 1). In summary, through direct comparison with both the *C. ljungdahlii* finished genome sequence and the Bruno-Barcena draft genome sequence, we can be confident that our Illumina sequence has called the correct bases in these instances.

A detailed review of the automated annotation of these proteins in the Brown et al*.* finished genome revealed that many of the discrepancies caused frame-shifts that resulted in premature or multiple stop codons to occur within the sequences (Table 1, Additional file 1). Moreover, the majority of these discrepancies (207 out of 243) occurred in homopolymer regions greater than five bases in length (Fig. 3) and the change present in each of these occurrences was the insertion of an additional monomer in our Illumina sequence, suggesting a tendency for calling strings of homopolymers short by PacBio technology at the time of publication of the Brown et al*.* finished genome sequence.

### Investigation of the origin reveals a previously undiscovered additional 181 base pair insertion

One identified discrepancy occurred at the beginning of the genome sequence assembly, where we observed a 1 base pair (bp) deletion. Investigation of this deletion by Sanger sequencing with primers ~350 bp upstream and downstream of the origin revealed a previously unidentified additional 181 bp. As neither the previous PacBio sequencing nor our own Illumina assembly revealed this insertion we performed a BLAST search of the region against *C. ljungdahlii* (GCA_000143685.1) to confirm its presence in the closely related acetogen. The start point for the assembly of *C. ljungdahlii* is in a different location to that of *C. autoethanogenum* finished genome sequences. The additional bases were present in *C. ljungdahlii* upstream of the *mopI* gene, which is in the same location as *C. autoethanogenum* relative to their CDS. The additional bases are in a non-coding region of the genome in both organisms.

### Manual annotation of our *C. autoethanogenum* finished genome provides a reliable reference for those working with this anaerobic acetogen

Our *C. autoethanogenum* finished sequence was uploaded to the genome annotation system GenDB [28], a user-friendly framework for genome assessment, annotation and curation. Annotation of the genome sequence was performed using GenDB version 2.4 [28]. Region prediction in the GenDB package is realized by the tools Prodigal [29] for coding sequences, tRNAScane-SE [30] for tRNAs and RNAMMER [31] for rRNAs. The Brown et al*. C. autoethanogenum* strain DSM 10061 finished genome sequence [16] was used as a reference for annotation with the following parameters e-value cut-off 10^−5^, with combined identity of 25 %, which means 50 % identity for 50 % of the length of the gene. This automatic annotation resulted in 3747 perfect matches, 73 matches with a different length.

Following automatic assignments, annotation of the identified ORFs was performed based on sequence similarity searches against sequence databases and subsequent manual curation and annotation using GenDB 2.4 [28]. Sequence similarity analyses were accomplished using blastx [26] against the NCBI non-redundant database on protein level [32], the Swissprot database [33, 34] and KEGG [35]. Additionally, manual gene annotation was performed using PRIAM [36], Motif Scan [37], Prosite [38], BRENDA [39, 40], UniProt/SwissProt [34], InterProScan [41], and Pfam [42] databases. One example of how our manual annotation differed from that of the automated pipeline used by Brown et al. can be found in the case of CLAU_3519 (CAETHG_3609). Here the automated pipeline from the Brown et al*.* finished genome assigned this gene product as a hypothetical protein, however when the sequence was aligned using BLASTP as part of our manual curation all other proteins with >75 % identity were named sodium ABC transporter. Upon further inspection in Pfam, one large ABC-2 family transporter protein domain was found (E-value 6.8e-31). Similar searches of UniProt and KEGG databases agreed with Pfam, therefore we annotated this gene product as an ABC-2 family transporter. The correction of the previously short-called homopolymer reads through our sequencing efforts gave a fully annotated finished sequence of *C. autoethanogenum* without the erroneous frame-shift containing annotations which had occurred previously.

Using these tools we were able to manually curate the entire genome to ensure that the automated annotation was correct and to insert additional information where required, as well as implementing a standardised protein product naming system as recommend by the NCBI guidelines [43] for ease of identification of genes with related functions. As a consequence of the automated and subsequent manual curation, we have found 482 instances across the genome where genes previously identified as ‘hypothetical protein’ have either been assigned a specific function, or have been named through identification of conserved domains based on sequence similarity. We have also encountered 131 instances where the annotation of a gene product in the Brown et al. finished sequence has been made less specific, or indeed reverted to ‘hypothetical protein’, as our searches have not been able to identify sufficient evidence to assign a specific function. The most common cause for the latter was previous identification of a gene product based on a minor region of coverage with homology to a distantly related species. Through comparison of the results of each protein sequence alignment to a broad selection of databases, we were able in some cases to correct and improve upon the automated annotation, highlighting the importance of manual curation where possible. This has supplied us with a robust and comprehensively annotated sequence for subsequent work with this industrially relevant acetogen (Table 2). The complete list of gene products with an altered function from those previously described in the Brown et al. genome annotation is provided in Additional file 3.

### Identification of coding sequences not previously detected in the Brown et al. genome annotation

Through application of the gene finding tool Prodigal, we have identified eight additional coding sequences which were not identified in the previous annotation, including one gene which has been annotated through sequence similarity as a M28 family peptidase (CLAU_1811), and one identified as a 3-oxoacyl-(acyl-carrier-protein) synthase 3 family protein (CLAU_2000). The following novel genes were all identified as ‘Hypothetical proteins’; CLAU_0723, CLAU_1503, CLAU_2529, CLAU_2784 and CLAU_3462. As a consequence of the correction of multiple frameshift mutations, many coding sequences previously annotated as two separate genes due to an erroneous stop codon have now been rectified into a single coding region, and as such our annotated genome now contains 3969 coding sequences, whereas the Brown et al. annotation at the time of publication contained 4042 coding sequences.

## Discussion

The current greatest technical challenge for creating single closed whole genome sequences is the presence of long stretches of repetitive DNA within those sequences, which hinders the assembly of shorter DNA reads into larger scaffolds and finished whole genome sequences. Many of the current technologies, including Illumina MiSeq, Ion Torrent and 454 GS FLX+ Titanium give read lengths in the region of 100–1000 base pairs, which compared with repetitive sequence lengths commonly found in bacteria of 5–7 Kb [44], is insufficient to create a single closed sequence without manual finishing, which can be costly and time-consuming.

The PacBio RS II sequencing system, used by Brown et al. [16] for generation of a closed WGS of *C. autoethanogenum*, was until recently the only long-read single-molecule sequencer available, and is capable of simplifying the process of genome assembly due to greatly increased read lengths [45]. Reads in excess of 15 Kb have been reported utilising the PacBio system [45], compared with Illumina MiSeq generating average read lengths of 250 base pairs in this study. Thus, the utilisation of PacBio systems for the generation of closed WGS’s from organisms that do not currently have such a sequence is highly advantageous in terms of both time and cost. However, it has been found that the error rate for PacBio sequencing is relatively high when compared to Illumina sequencing data [46, 47], especially concerning homopolymer regions between two and fourteen base pairs in length [48]. In our study, we demonstrated a heavy bias towards under-calling of homopolymer regions, which in this example led to ~240 erroneous deletions from the ~4.35 Mb genome of *C. autoethanogenum*. This high error rate is in-line with previous findings on long-read assemblies [45], and in recent years improvements to the algorithms used by PacBio have had the consequence of reducing the overall error rate significantly. However, it may still be the case that the PacBio system should ideally be used in conjunction with other forms of sequencing following PacBio assembly, such as Illumina MiSeq and Sanger sequencing, to ensure accuracy of the data, certainly for assemblies performed with earlier iterations of the PacBio technology, as is the case with the dataset in question here. The recently released Oxford Nanopore technology has potential to further revolutionise the field of genome sequencing over the coming years, allowing label-free, ultra-long reads (10^4^–10^6^ bases), with the capability for extremely high throughput, and low material requirement [49].

## Conclusions

The whole genome sequence of *C. autoethanogenum* presented here-in represents a correction of the sequencing errors present in the previously published closed genome sequence generated primarily from an early iteration of PacBio sequencing technology. It was annotated via an automated pipeline and further curated manually to ensure the quality of annotation. This has resulted in the generation of the most accurate closed-genome sequence of the industrially relevant acetogen *C. autoethanogenum* to date and is an important step forward for academic institutions and industrial companies that wish to study and / or manipulate this organism for the purposes of high-value chemical production.

## Methods

### Bacterial growth and DNA isolation

The *C. autoethanogenum* JA1-1 strain was obtained as a freeze-dried stock from the Deutsche Sammlung von Mikroorganismen und Zellkulturen GmbH (DSMZ) culture collection (DSM 10061) and revived by growth on a YTF agar medium (per L; Yeast extract 10 g, tryptone 16 g, fructose 10 g, Na chloride 0.2 g, 1000× acidic trace element solution 1 ml (per L; 50 mM HCl, H_3_BO_3_ 100 mg, MnCl_2_.4H_2_O 230 mg, FeCl_2_.4H_2_O 780 mg, CoCl_2_.6H_2_O 103 mg, NiCl_2_.6H_2_O 602 mg, ZnCl_2_ 78 mg, CuSO_4_.5H_2_O 50 mg, AlK(SO_4_)_2_.12H_2_O 50 mg), 1000× basic trace element solution 1 ml (per L; NaOH 10 mM, Na_2_SeO_3_ 58 mg, Na_2_WO_4_ 53 mg, Na_2_MbO_4_.2H_2_O 52 mg), 1000× vitamin solution 1 ml (per 500 ml; p-aminobenzoate 57 mg, riboflavin 52 mg, thiamine 100 mg, nicotinate 103, pyridoxine 255 mg, Ca D-(+)-pantothenate 52 mg, cyanocobalamin 39 mg, d-biotin 11 mg, folate 24 mg, thioctic acid 25 mg), agar 15 g, pH 5.8) in an anaerobic cabinet (Don Whitley) at 37 °C. For storage and DNA isolation, the strain was sub-cultured into liquid YTF medium and grown to mid-exponential phase prior to harvesting. Samples were stored in 25 % glycerol at −80 °C. Genomic DNA isolation was by phenol:chloroform extraction based on the method of Mamur [50]. Genomic DNA was quantified with a NanoDrop ND-1000 spectrophotometer (Labtech International) and the quality was determined via agarose gel electrophoresis. Whole genome sequencing was performed using an Illumina MiSeq instrument in the DeepSeq facility at the University of Nottingham. Sequencing data was mapped against the published *C. autoethanogenum* sequence available in the NCBI database (GenBank: CP006763) using the program CLC Genomics Workbench (CLC Bio; Qiagen).

### Genome sequencing data generation

Genome sequencing was achieved at the DeepSeq next generation sequencing facility at the University of Nottingham. Samples were sequenced using an Illumina MiSeq desktop sequencer, a paired-end approach was taken with reads lengths of 250 base pairs.

### Sequencing data trimming, filtering and assembly

Illumina mate-paired reads were trimmed of their adaptor sequences and filtered for quality using the program CLC Genomics Workbench (v. 7.0.4, CLC bio, Denmark), and subsequently assembled using DSM10061 as a reference sequence (Additional file 4).

### PCR and Sanger sequencing

*C. autoethanogenum* genomic DNA was used as a template for PCR reactions using Q5 High-Fidelity DNA Polymerase (New England Biolabs Inc.) as per manufacturer’s instructions with primers specific for the region to be sequenced (see Additional file 5). PCR products were analysed by agarose gel electrophoresis and DNA recovered using the Zymoclean™ Gel DNA Recovery Kit available from Zymo Research with elution into 6 μl sterile water. Samples were sent for Sanger sequencing at Source BioScience LifeSciences with the appropriate primer(s).

### Automated annotation pipeline tools

The automated pipeline for annotation was performed using the software package GenDB version 2.4 [28]. Region prediction in the GenDB package is realized by the tools Prodigal version 2.6.0 [29] for coding sequences, tRNAScane-SE version 1.21 [30] for tRNAs and RNAMMER version 1.2 [31] for rRNAs.

### Availability of supporting data

The whole genome data sets supporting the results of this article are available in the National Center for Biotechnology Information (NCBI) repository, accession number CP012395, and the raw Illumina data available within the NCBI Sequence Read Archive under accession number SRP066900. Sanger sequencing trace data is available upon request. All other data sets supporting the results of this article are included within the article (and its additional files).

## Additional files

Additional file 1:
**Discrepancies occurring between the current and Brown et al. finished genome sequence of** ***C. autoethanogenum.*** This table shows all of the discrepancies that occur when our finished genome sequence (CLAU) is mapped against the Brown et al. finished genome sequence (BRO). Mutation column describes the mutation occurring in the CLAU genome compared to the BRO genome. Gene / region gives the gene name where the discrepancy occurs, ← / ← or similar denotes that the discrepancy occurred in a non-coding region between the named genes. Homopolymer length indicates the number of the same base occurring consecutively at the site of the discrepancy. Amino acid length gives the annotated protein length of the gene in which the discrepancy occurs, *indicates protein codes for multiple stop codons and ^indicates that no stop codon was found in the annotation. The sequence identity is relative to the CLAU *C. autoethanogenum* genome sequence when protein BLAST searched on the NCBI database. CLAU, *C. autoethanogenum* finished genome sequence in present study; CLJU, *C. ljungdahlii* DSM 13528 finished genome sequence (GCA_000143685.1); BRO, Brown et al*. C. autoethanogenum* finished genome sequence (GCA_000484505.1); CAUT, Bruno-Barcena et al. *C. autoethanogenum* draft genome sequence (GCA_000427255.1); NF, not found. (DOCX 73 kb) Additional file 2:
**Sanger sequencing of selected discrepancies between the current and Brown et al. sequences.** Table showing the region around the discrepancies between our finished genome sequence, confirmed by Sanger sequencing, and the Brown et al. finished genome sequence. (DOCX 23 kb) Additional file 3:
**Complete list of gene products with an alternative function to that previously described by Brown et al.** (XLSX 101 kb) Additional file 4:
**Illumina mapping summary report.** (DOCX 84 kb) Additional file 5:
**List of primers used in this study. A list of a forward and reverse primers used in this study for verification of whole genome sequencing.** (DOCX 17 kb)

## Abbreviations

YTF: yeast tryptone fructose
NCBI: National Center for Biotechnology Information
WGS: whole genome sequence
BLAST: basic local alignment search tool
CDS: coding sequence(s)
