# Supplementary material for: Whole genome sequence and manual annotation of Clostridium autoethanogenum, an industrially relevant bacterium
Source: BMC Genomics. 2015 Dec 21;16:1085. doi: 10.1186/s12864-015-2287-5 (PMC4687164; doi:10.1186/s12864-015-2287-5)
Supplement: Additional file 1: — Discrepancies occurring between the current and Brown et al. finished genome sequence of C. autoethanogenum. This table shows all of the discrepancies that occur when our finished genome sequence (CLAU) is mapped against the Brown et al. finished genome sequence (BRO). Mutation column describes the mutation occurring in the CLAU genome compared to the BRO genome. Gene / region gives the gene name where the discrepancy occurs, ← / ← or similar denotes that the discrepancy occurred in a non-coding region between the named genes. Homopolymer length indicates the number of the same base occurring consecutively at the site of the discrepancy. Amino acid length gives the annotated protein length of the gene in which the discrepancy occurs, *indicates protein codes for multiple stop codons and ^indicates that no stop codon was found in the annotation. The sequence identity is relative to the CLAU C. autoethanogenum genome sequence when protein BLAST searched on the NCBI database. CLAU, C. autoethanogenum finished genome sequence in present study; CLJU, C. ljungdahlii DSM 13528 finished genome sequence (GCA_000143685.1); BRO, Brown et al. C. autoethanogenum finished genome sequence (GCA_000484505.1); CAUT, Bruno-Barcena et al. C. autoethanogenum draft genome sequence (GCA_000427255.1); NF, not found. (DOCX 73 kb) [file 12864_2015_2287_MOESM1_ESM.docx]

**Additional file 1: Discrepancies occurring between the current and Brown *et al*. whole genome sequencing of *C. autoethanogenum***

| Position | Mutation | Gene / region | Homopolymer length | | Amino acid length | | Sequence identity | | |
| --- | --- | --- | --- | --- | --- | --- | --- | --- | --- |
|  |  |  | CLAU | CLJU | CLAU | BRO | BRO | CAUT | CLJU |
| 1 | -A | → CAETHG_0001 | 1 | 1 | - | - | - | - | - |
| 3259 | A | CAETHG_0005 | 6 | 6 | 409 | 299 | 293/293 | 407/408 | 407/408 |
| 29841 | T | CAETHG_0036 | 5 | 5 | 392 | 368 | 364/364 | NF | 392/392 |
| 44272 | T | CAETHG_0050 | 7 | 7 | 359 | 323 | 315/315 | NF | 358/359 |
| 45316 | G | CAETHG_0050 ← / → CAETHG_0051 | 6 | 6 | - | - | - | - | - |
| 46129 | T | CAETHG_0051 | 6 | 6 | 412 | 412 | 119/367 | NF | 412/412 |
| 47240 | T | CAETHG_0053 | 6 | 6 | 412 | 116 | 119/367 | NF | 412/412 |
| 66496 | T | CAETHG_0070 → / → CAETHG_0071 | 8 | 8 | - | - | - | - | - |
| 117478 | A | CAETHG_0113 | 7 | 7 | 1164 | 778 | 776/776 | NF | 1147/1164 |
| 124794 | A | CAETHG_0119 | 7 | 7 | 638 | 447 | 428/428 | NF | 638/638 |
| 127144 | A | CAETHG_0121 → / → CAETHG_0122 | 7 | 7 | - | - | - | - | - |
| 127389 | A | CAETHG_0121 → / → CAETHG_0122 | 7 | 7 | - | - | - | - | - |
| 127400 | A | CAETHG_0121 → / → CAETHG_0122 | 7 | 7 | - | - | - | - | - |
| 127486 | T | CAETHG_0121 → / → CAETHG_0122 | 5 | 5 | - | - | - | - | - |
| 159945 | G | CAETHG_0150 | 5 | 5 | 208 | 206 | 206/206 | NF | 208/208 |
| 161225 | A | CAETHG_0152 | 8 | 8 | 265 | 41 | 216/216 | NF | 265/265 |
| 163952 | T | CAETHG_0155 → / ← CAETHG_0156 | 7 | 7 | - | - | - | - | - |
| 165377 | T | CAETHG_0156 ← / ← CAETHG_0157 | 8 | 8 | - | - | - | - | - |
| 165391 | T | CAETHG_0157 | 5 | 5 | 617 | 141 | 140/140 + 464/464 | 617/617 | 590/617 |
| 168124 | G | CAETHG_0159 | 5 | 5 | 211 | 184 | 174/174 | NF | 211/211 |
| 172464 | G | CAETHG_0163 ← / → CAETHG_0164 | 2 | 2 | - | - | - | - | - |
| 174241 | T | CAETHG_0165 | 6 | 6 | 618 | 483 | 478/483 | NF | 618/618 |
| 182488 | T | CAETHG_0172 → / → CAETHG_0173 | 4 | 4 | - | - | - | - | - |
| 190962 | G | CAETHG_0183 ← / ← CAETHG_0184 | 4 | 4 | - | - | - | - | - |
| 238048 | A | CAETHG_0227 ← / → CAETHG_0228 | 7 | 7 | - | - | - | - | - |
| 251180 | A | CAETHG_0235 | 8 | 8 | 1010 | 603 | 597/597 | NF | 1010/1010 |
| 280934 | C | CAETHG_0260 ← / ← CAETHG_0261 | 6 | 6 | - | - | - | - | - |
| 282548 | C | CAETHG_0262 | 5 | 5 | 289 | 268 | 261/261 | NF | 289/289 |
| 283331 | C | CAETHG_0263 | 5 | 5 | 370 | 370 | NF | 370/370 | 369/370 |
| 287229 | T | CAETHG_0269 | 8 | 8 | 302 | 202 | 192/194 | NF | 249/249 |
| 314151 | C | CAETHG_0298 | 4 | 4 | 570 | 555 | 553/553 | 570/570 | 569/570 |
| 338255 | G | CAETHG_0319 → / ← CAETHG_0320 | 5 | 5 | - | - | - | - | - |
| 342673 | A | CAETHG_0326 | 7 | 7 | 247 | 143 | 143/143 | NF | 247/247 |
| 348516 | T | CAETHG_0332 | 6 | 6 | 312 | 201 | 193/201 | NF | 312/312 |
| 389079 | C | CAETHG_0363 | 5 | 5 | 402 | 402 | NF | 402/402 | 398/402 |
| 423998 | T | CAETHG_0392 | 6 | 6 | 1046 | 277 | NF | NF | 1046/1046 |
| 465361 | C | CAETHG_0425 ← / ← CAETHG_0426 | 4 | 4 | - | - | - | - | - |
| 475299 | C | CAETHG_0435 | 4 | 4 | 407 | 370 | 363/363 | 407/407 | 400/407 |
| 479940 | A | CAETHG_0439 | 7 | 7 | 430 | 105 | 101/104 | 430/430 | 425/430 |
| 482992 | A | CAETHG_0442 ← / ← CAETHG_0443 | 6 | 6 | - | - | - | - | - |
| 514320 | C | CAETHG_0463 ← / ← CAETHG_0464 | 7 | 7 | - | - | - | - | - |
| 547091 | T | CAETHG_0490 | 6 | 6 | 339 | 281 | 266/269 | NF | 339/339 |
| 552005 | T | CAETHG_0494 | 7 | 7 | 511 | 205 | 200/203 + 306/307 | 511/511 | 431/431 |
| 559107 | T | CAETHG_0499 ← / ← CAETHG_0500 | 5 | 5 | - | - | - | - | - |
| 576961 | A | CAETHG_0519 → / ← CAETHG_0520 | 2 | 2 | - | - | - | - | - |
| 613244 | A | CAETHG_0554 | 7 | 7 | 632 | 641 | 626/631 | NF | 632/632 |
| 617697 | C | CAETHG_0557 ← / ← CAETHG_0558 | 6 | 6 | - | - | - | - | - |
| 627984 | C | CAETHG_0567 | 2 | 2 | 521 | 245 | 231/233 | NF | 521/521 |
| 656810 | T | CAETHG_0595 | 6 | 6 | 722 | 279 | 269/269 | 722/722 | 717/722 |
| 693187 | T | CAETHG_0632 | 8 | 8 | 125 | 42 | 35/42 | NF | 125/125 |
| 755993 | T | CAETHG_0689 | 8 | 8 | 378 | 238 | 230/233 | 378/378 | NF |
| 757677 | T | CAETHG_0691 | 6 | 6 | 891 | 572 | 563/563 | NF | NF |
| 776817 | C | CAETHG_0714 | 6 | 6 | 313 | 196 | 182/182 | NF | 313/313 |
| 786900 | A | CAETHG_0728 | 8 | 8 | 370 | 358 | 357/357 | 370/370 | 370/370 |
| 793985 | T | CAETHG_0734 ← / → CAETHG_0735 | 5 | 5 | - | - | - | - | - |
| 826486 | T | CAETHG_0764 | 7 | 7 | 373 | 280 | NF | 372/373 | 368/373 |
| 826929 | A | CAETHG_0764, CAETHG_0766 | 8 | 8 | 373 | 278^ + 107 | 105/107 | 361/362 | 367/373 |
| 846450 | T | CAETHG_0790 | 5 | 5 | 386 | 121 | 109/110 | NF | 386/386 |
| 859894 | G | CAETHG_0803 | 6 | 6 | 228 | 553 | 223/224 | NF | 228/228 |
| 866415 | C | CAETHG_0808 | 6 | 6 | 431 | 300 | 297/298 | NF | 431/431 |
| 902073 | T | CAETHG_0837 | 7 | 7 | 237 | 75 | 75/75 | 236/237 | 236/237 |
| 919361 | A | CAETHG_0852 | 7 | 7 | 505 | 413 | 401/402 | NF | 505/505 |
| 928129 | C | CAETHG_0862 | 5 | 5 | 293 | 250 | 249/249 | NF | 293/293 |
| 945907 | T | CAETHG_0877 | 7 | 7 | 442 | 82 | 82/82 + 342/343 | NF | 442/442 |
| 947320 | T | CAETHG_0878 ← / ← CAETHG_0879 | 8 | 8 | - | - | - | - | - |
| 967462 | A | CAETHG_0898 ← / ← CAETHG_0899 | 7 | 7 | - | - | - | - | - |
| 985484 | C | CAETHG_0915 | 4 | 4 | 688 | 688 | NF | NF | 688/688 |
| 992036 | C | CAETHG_0922 | 6 | 6 | 266 | 208 | 204/204 | NF | 266/266 |
| 1031822 | T | CAETHG_0963 | 7 | 7 | 192 | 99 | 94/94 | 192/192 | 192/192 |
| 1038572 | G | CAETHG_0970 | 5 | 5 | 460 | 104 | 97/107 | 458/458 | 459/460 |
| 1080750 | A | CAETHG_1007 | 7 | 7 | 460 | 308 | 307/307 | 460/460 | 456/460 |
| 1098251 | T | CAETHG_1021 ← / ← CAETHG_1022 | 7 | 7 | - | - | - | - | - |
| 1099831 | T | CAETHG_1024 | 8 | 8 | 222 | 152 | 146/146 | 222/222 | NF |
| 1106176 | A | CAETHG_1030 | 6 | 6 | 172 | 126 | 109/109 | NF | 172/172 |
| 1124614 | T | CAETHG_1042 ← / → CAETHG_1043 | 7 | NF | - | - | - | - | - |
| 1141473 | T | CAETHG_1056 | 6 | 6 | 224 | 204 | 204/204 | NF | 224/224 |
| 1147446 | T | CAETHG_1062 | 6 | 6 | 571 | 373 | 372/372 | 571/571 | 569/571 |
| 1148633 | T | CAETHG_1062 ← / ← CAETHG_1063 | 7 | 7 | - | - | - | - | - |
| 1177671 | T | CAETHG_1086 ← / ← CAETHG_1087 | 6 | 6 | - | - | - | - | - |
| 1184449 | T | CAETHG_1096 | 6 | 6 | 301 | 136 | 131/130 | 301/301 | NF |
| 1250270 | A | CAETHG_1165 → / → CAETHG_1166 | 8 | 8 | - | - | - | - | - |
| 1269509 | A | CAETHG_1186 ← / → CAETHG_1187 | 7 | 7 | - | - | - | - | - |
| 1304577 | G | CAETHG_1218 → / ← CAETHG_1219 | 6 | 6 | - | - | - | - | - |
| 1307050 | T | CAETHG_1221 | 6 | 6 | 327 | 360 | 319/319 | NF | 327/327 |
| 1329519 | C | CAETHG_1246 ← / → CAETHG_1247 | 6 | 6 | - | - | - | - | - |
| 1359415 | C | CAETHG_1270 | 6 | 6 | 172 | 182 | 171/172 | NF | 172/172 |
| 1364936 | T | CAETHG_1277 | 8 | 8 | 108 | 110 | 106/107 | NF | 107/108 |
| 1408410 | T | CAETHG_1317 | 2 | 2 | 612 | 540 | 540/540 | NF | 612/612 |
| 1421430 | G | CAETHG_1330 | 5 | 5 | 407 | 299 | 292/299 | NF | 407/407 |
| 1446223 | T | CAETHG_1349 ← / → CAETHG_1350 | 5 | 5 | - | - | - | - | - |
| 1457002 | C | CAETHG_1363 | 6 | 6 | 296 | 254 | 249/249 | 294/295 | 292/296 |
| 1486496 | T | CAETHG_1391 ← / ← CAETHG_1392 | 8 | 8 | - | - | - | - | - |
| 1512419 | G | CAETHG_1413 → / → CAETHG_1414 | 5 | 5 | - | - | - | - | - |
| 1518063 | C | CAETHG_1420 ← / ← CAETHG_1421 | 4 | 4 | - | - | - | - | - |
| 1518678 | C | CAETHG_1421 | 6 | 6 | 106 | 279 | 105/105 | 106/106 | 105/106 |
| 1519116 | A | CAETHG_1421 ← / → CAETHG_1422 | 10 | 10 | - | - | - | - | - |
| 1564306 | C | CAETHG_1461 ← / ← CAETHG_1462 | 5 | 5 | - | - | - | - | - |
| 1600445 | T | CAETHG_1498 | 7 | 7 | 413 | 422 | 407/410 | NF | 413/413 |
| 1603900 | T | CAETHG_1501 | 8 | 8 | 401 | 401 | NF | NF | 401/401 |
| 1609463 | T | CAETHG_1508 | 7 | 7 | 251 | 247 | 242/242 | 250/250 | 251/251 |
| 1620246 | T | CAETHG_1521 | 6 | NF | 323 | 316 | 315/315 | 323/323 | 310/323 |
| 1627977 | T | CAETHG_1529 ← / → CAETHG_1530 | 6 | NF | - | - | - | - | - |
| 1628459 | A | CAETHG_1530 | 4 | 4 | 570 | 78 | NF | 570/570 | 567/570 |
| 1724767 | C | CAETHG_1601 | 6 | CACCCC | 280 | 280 | NF | 280/280 | 271/280 |
| 1794958 | G | CAETHG_1672 ← / → CAETHG_1673 | 5 | 5 | - | - | - | - | - |
| 1801153 | T | CAETHG_1679 ← / → CAETHG_1680 | 7 | 7 | - | - | - | - | - |
| 1818816 | C | CAETHG_1690 | 5 | 5 | 400 | 241 | 241/241 | 400/400 | 398/400 |
| 1840643 | G | CAETHG_1706 → / → CAETHG_1707 | 5 | 5 | - | - | - | - | - |
| 1841888 | T | CAETHG_1708 → / → CAETHG_1709 | 2 | 2 | - | - | - | - | - |
| 1842972 | A | CAETHG_1710 | 6 | 6 | 537 | 96 | 90/95 | 537/537 | NF |
| 1854392 | T | CAETHG_1719 | 6 | 6 | 748 | 748* | NF | 735/735 | 726/745 |
| 1855004 | T | CAETHG_1721 | 7 | 7 | 127 | 140 | 125/126 | 127/127 | 126/127 |
| 1904617 | C | CAETHG_1767 ← / ← CAETHG_1768 | 5 | 5 | - | - | - | - | - |
| 1909195 | G | CAETHG_1772 | 4 | 4 | 214 | 63 | 61/63 | NF | 214/214 |
| 1979822 | C | CAETHG_1841 | 5 | 5 | 397 | 405 | 392/394 | NF | 388/388 |
| 1983427 | T | CAETHG_1846 | 7 | 7 | 663 | 405 | 404/405 | NF | 663/663 |
| 1993028 | C | CAETHG_1856 ← / ← CAETHG_1857 | 5 | 5 | - | - | - | - | - |
| 2066166 | T | CAETHG_1922 | 7 | 7 | 132 | 103 | NF | NF | 132/132 |
| 2099300 | C | CAETHG_1967 ← / ← CAETHG_1968 | 4 | 4 | - | - | - | - | - |
| 2134918 | T | CAETHG_1990 ← / ← CAETHG_R0038 | 9 | 9 | - | - | - | - | - |
| 2147004 | C | CAETHG_2001 ← / ← CAETHG_2002 | 8 | 8 | - | - | - | - | - |
| 2154269 | T | CAETHG_2008 ← / ← CAETHG_2009 | 6 | 6 | - | - | - | - | - |
| 2162064 | G | CAETHG_2018 | 4 | 4 | 310 | 274 | 269/269 | NF | 310/310 |
| 2170478 | G | CAETHG_2024 → / ← CAETHG_2025 | 4 | 4 | - | - | - | - | - |
| 2174475 | A | CAETHG_2030 | 7 | 7 | 166 | 67 | 57/67 + 102/102 | NF | 166/166 |
| 2180132 | G | CAETHG_2036 → / → CAETHG_2037 | 5 | 5 | - | - | - | - | - |
| 2190231 | C | CAETHG_2048 | 6 | 6 | 312 | 190 | 186/186 + 110/110 | NF | 312/312 |
| 2222019 | T | CAETHG_2078 | 8 | 8 | 445 | 326 | 325/325 | NF | 444/445 |
| 2238733 | A | CAETHG_R0029 ← / → CAETHG_2094 | 8 | 8 | - | - | - | - | - |
| 2246483 | T | CAETHG_2100 | 8 | 8 | 328 | 327* | NF | NF | 328/328 |
| 2266467 | A | CAETHG_2127 | 8 | 8 | 366 | 348 | 347/347 | NF | 366/366 |
| 2277544 | A | CAETHG_2131 → / → CAETHG_2132 | 8 | 8 | - | - | - | - | - |
| 2281989 | A | CAETHG_2134 | 8 | 8 | 463 | 462* | NF | NF | 463/463 |
| 2313962 | T | CAETHG_2170 | 2 | 2 | 250 | 231 | 219/220 | NF | 250/250 |
| 2338199 | C | CAETHG_2193 | 6 | 6 | 247 | 249 | 242/246 | NF | 247/247 |
| 2352969 | T | CAETHG_2212, CAETHG_2213 | 2 | 2 | 416 | 202 + 641 | 198/200 | 416/416 | 414/416 |
| 2384248 | G | CAETHG_2231 | 6 | 6 | 361 | 191 | 189/190 + 142/142 | NF | 361/361 |
| 2423101 | G | CAETHG_2266 → / → CAETHG_2267 | 6 | 6 | - | - | - | - | - |
| 2435091 | A | CAETHG_2280 | 6 | 6 | 434 | 59 | 55/57 + 374/374 | NF | 433/434 |
| 2443036 | C | CAETHG_2289 → / ← CAETHG_2290 | 6 | 6 | - | - | - | - | - |
| 2459242 | T | CAETHG_2299 → / → CAETHG_2300 | 7 | 7 | - | - | - | - | - |
| 2463469 | A | CAETHG_2302 ← / → CAETHG_2303 | 5 | 5 | - | - | - | - | - |
| 2463958 | A | CAETHG_2303 | 6 | 6 | 335 | 128 | 127/127 + 207/207 | NF | 334/335 |
| 2464961 | C | CAETHG_2305 → / → CAETHG_2306 | 1 | 1 | - | - | - | - | - |
| 2465456 | A | CAETHG_2306 | 5 | 5 | 255 | 168 | 159/159 + 45/45 | NF | 254/255 |
| 2505955 | A | CAETHG_2347 | 6 | 6 | 504 | 484 | 478/480 | NF | 504/504 |
| 2511280 | T | CAETHG_2352 → / → CAETHG_2353 | 7 | 7 | - | - | - | - | - |
| 2520865 | T | CAETHG_2360 | 3 | 3 | 340 | 256 | 248/253 + 66/67 | NF | 340/340 |
| 2535012 | T | CAETHG_2371 | 7 | 7 | 612 | 189 | 185/187 + 407/407 | 611/612 | 599/612 |
| 2538245 | A | CAETHG_2375 ← / → CAETHG_2376 | 7 | 7 | - | - | - | - | - |
| 2549593 | A | CAETHG_2385 → / ← CAETHG_2386 | 6 | NF | - | - | - | - | - |
| 2558591 | A | CAETHG_2394 | 8 | NF | 399 | 49 | 48/49 + 336/337 | 399/399 | NF |
| 2562954 | A | CAETHG_2399 | 3 | NF | 574 | 132 | 100/100 + 444/444 | 574/574 | NF |
| 2596835 | G | CAETHG_2429 | 7 | 7 | 400 | 382 | 377/378 | NF | 399/400 |
| 2597158 | G | CAETHG_2429 → / → CAETHG_2430 | 7 | 7 | - | - | - | - | - |
| 2611540 | A | CAETHG_2440 | 7 | 7 | 585 | 60 | 52/53 + 522/522 | NF | 585/585 |
| 2633450 | G | CAETHG_2459 | 4 | 4 | 1018 | 1017* | NF | **649/650 + 367/367** | 960/1018 |
| 2666469 | A | CAETHG_2486 → / → CAETHG_2487 | 6 | 6 | - | - | - | - | - |
| 2671182 | G | CAETHG_2492 → / → CAETHG_2493 | 5 | 5 | - | - | - | - | - |
| 2683087 | C | CAETHG_2503 | 4 | 4 | 640 | 615 | 601/605 | 640/640 | 639/640 |
| 2689105 | A | CAETHG_2507 ← / → CAETHG_2508 | 8 | 8 | - | - | - | - | - |
| 2689940 | A | CAETHG_2508 | 6 | 6 | 349 | 235 | 234/235 + 98/99 | 349/349 | 344/349 |
| 2766440 | A | CAETHG_2569 → / → CAETHG_2570 | 7 | 7 | - | - | - | - | - |
| 2779608 | G | CAETHG_2576 → / ← CAETHG_2577 | 5 | 5 | - | - | - | - | - |
| 2804423 | T | CAETHG_2599 | 8 | 8 | 372 | 318 | 310/314 + 56/57 | 372/372 | 368/372 |
| 2805023 | A | CAETHG_2601, CAETHG_2602 | 7 | AAAGAAA | 370 | 141 + 244 | 138/138 + 234/234 | 370/370 | 328/366 |
| 2815868 | A | CAETHG_2612, CAETHG_2613 | 6 | NF | 376 | 71 + 313 | 66/69 + 311/313 | 376/376 | NF |
| 2823726 | C | CAETHG_2620 | 5 | 5 | 513 | 512* | NF | 361/361 + 89/90 | 508/513 |
| 2827089 | A | CAETHG_2624 | 8 | 8 | 371 | 59 | 46/50 + 247/247 | 371/371 | 370/371 |
| 2827314 | A | CAETHG_2624 → / → CAETHG_2625 | 8 | 8 | - | - | - | - | - |
| 2840808 | G | CAETHG_2636 | 5 | 5 | 455 | 202 | 186/186 + 260/260 | NF | 455/455 |
| 2852812 | T | CAETHG_2647 | 8 | NF | 470 | 314 | 314/314 + 123/124 | 470/470 | NF |
| 2874342 | T | CAETHG_2664, CAETHG_2665 | 2 | 2 | 781 | 602 + 192 | 593/601 + 11/192 | NF | 781/781 |
| 2894334 | G | CAETHG_2686 | 5 | 5 | 1111 | 351 | 345/345 + 686/687 | 586/586 | 1100/1111 |
| 2898108 | C | CAETHG_2688 ← / ← CAETHG_2689 | 5 | 5 | - | - | - | - | - |
| 2909240 | G | CAETHG_2700 → / → CAETHG_2701 | 5 | 5 | - | - | - | - | - |
| 2923771 | A | CAETHG_2715 | 7 | 7 | 420 | 90 | 89/89 + 269/269 | 420/420 | 418/420 |
| 2940344 | A | CAETHG_R0076 → / → CAETHG_2723 | 7 | 7 | - | - | - | - | - |
| 2943659 | G | CAETHG_2725 → / → CAETHG_2726 | 5 | 5 | - | - | - | - | - |
| 2948984 | T | CAETHG_2732 → / → CAETHG_2733 | 7 | 7 | - | - | - | - | - |
| 2977137 | A | CAETHG_2751 | 8 | 8 | 453 | 460 | 448/453 | NF | 453/453 |
| 2988745 | G | CAETHG_2760 | 4 | 4 | 401 | 62 | 55/57 + 333/334 | NF | 401/401 |
| 2996548 | A | CAETHG_2768 → / → CAETHG_2769 | 6 | 6 | - | - | - | - | - |
| 3042834 | A | CAETHG_2803 | 7 | 7 | 613 | 457 | 452/453 + 124/125 | NF | 613/613 |
| 3045051 | G | CAETHG_2806 → / → CAETHG_2807 | 5 | 5 | - | - | - | - | - |
| 3048887 | G | CAETHG_2810 → / → CAETHG_2811 | 5 | 5 | - | - | - | - | - |
| 3076804 | A | CAETHG_2840 | 8 | 8 | 635 | 487 | 480/483 + 147/147 | NF | 635/635 |
| 3097545 | A | CAETHG_2860 → / → CAETHG_2861 | 7 | 7 | - | - | - | - | - |
| 3114090 | T | CAETHG_2873 | 8 | 8 | 308 | 307* | NF | 308/308 | 307/308 |
| 3138531 | A | CAETHG_2894 | 8 | 8 | 251 | 180 | 180/180 + 54/55 | NF | 251/251 |
| 3148223 | A | CAETHG_2906 | 7 | 7 | 257 | 227 | 222/226 | NF | 256/257 |
| 3154595 | G | CAETHG_2911 | 6 | 6 | 396 | 364 | 359/361 | 396/396 | 394/396 |
| 3158197 | T | CAETHG_2915 ← / → CAETHG_2916 | 8 | 8 | - | - | - | - | - |
| 3158220 | T | CAETHG_2915 ← / → CAETHG_2916 | 5 | 5 | - | - | - | - | - |
| 3160180 | A | CAETHG_2916 → / → CAETHG_2917 | 7 | 7 | - | - | - | - | - |
| 3161184 | A | CAETHG_2917 | 6 | 6 | 361 | 319 | 318/319 | 354/354 | 354/354 |
| 3201513 | A | CAETHG_2958 | 9 | 9 | 107 | 146 | 106/106 | NF | 107/107 |
| 3220068 | T | CAETHG_2973 | 8 | 8 | 250 | 249* | NF | 250/250 | 249/250 |
| 3238858 | G | CAETHG_2988 → / → CAETHG_2989 | 5 | 5 | - | - | - | - | - |
| 3265817 | G | CAETHG_3010 → / → CAETHG_3011 | 6 | 6 | - | - | - | - | - |
| 3283928 | G | CAETHG_3023 | 5 | 5 | 487 | 206 | 203/204 + 274/274 | 475/476 | 486/487 |
| 3313542 | G | CAETHG_3050 → / → CAETHG_3051 | 6 | 6 | - | - | - | - | - |
| 3376286 | G | CAETHG_3108 → / → CAETHG_3109 | 7 | 7 | - | - | - | - | - |
| 3396986 | G | CAETHG_3132, CAETHG_3133 | 5 | 5 | 160 | 152 + 80 | 149/149 | NF | 160/160 |
| 3410030 | A | CAETHG_3142 ← / → CAETHG_3143 | 8 | 8 | - | - | - | - | - |
| 3467738 | G | CAETHG_3210 | 4 | 4 | 368 | 97 | 85/87 + 275/276 | NF | 368/368 |
| 3468796 | G | CAETHG_3212 | 5 | 5 | 271 | 291 | 270/271 | NF | 270/271 |
| 3468964 | C → A | CAETHG_3212 | 2 | 1 | 271 | 291 | 270/271 | NF | 270/271 |
| 3477442 | A | CAETHG_3223 ← / → CAETHG_3224 | 2 | 2 | - | - | - | - | - |
| 3505018 | T | CAETHG_3250 → / → CAETHG_3251 | 6 | 6 | - | - | - | - | - |
| 3562615 | A | CAETHG_3308 | 8 | 8 | 233 | 220 | 218/219 | NF | 232/233 |
| 3585477 | C | CAETHG_3330 → / → CAETHG_3331 | 4 | 4 | - | - | - | - | - |
| 3610061 | T | CAETHG_3356 | 9 | 9 | 388 | 127 | 126/127 + 232/232 | NF | 387/388 |
| 3630543 | A | CAETHG_3380 ← / ← CAETHG_3381 | 7 | 7 | - | - | - | - | - |
| 3673040 | A | CAETHG_3421 | 7 | 7 | 310 | 67 | 62/63 + 183/184 | 309/310 | 307/310 |
| 3724084 | T | CAETHG_3470 ← / → CAETHG_3471 | 8 | 8 | - | - | - | - | - |
| 3741209 | T | CAETHG_3490 | 7 | 7 | 405 | 337 | 334/334 | NF | 405/405 |
| 3752592 | G | CAETHG_3500 | 5 | 5 | 459 | 418 | 413/415 + 41/41 | NF | 459/459 |
| 3767586 | A | CAETHG_3514 → / → CAETHG_3515 | 6 | 6 | - | - | - | - | - |
| 3783959 | T | CAETHG_3526 | 5 | NF | 568 | 405 | 404/405 + 156/157 | 568/568 | NF |
| 3786709 | T | CAETHG_3531 | 6 | NF | 144 | 64 | 64/64 + 79/80 | 144/144 | NF |
| 3787895 | T | CAETHG_3534 | 3 | NF | 348 | 230 | 229/229 + 106/106 | 348/348 | NF |
| 3811864 | G | CAETHG_3558 | 5 | NF | 103 | 112 | 91/93 | 103/103 | NF |
| 3827441 | T | CAETHG_3567 | 3 | 3 | 231 | 98 | 81/95 + 133/134 | NF | 230/231 |
| 3848200 | A | CAETHG_3579 ← / → CAETHG_3580 | 4 | NF | - | - | - | - | - |
| 3877937 | A | CAETHG_3599 | 3 | 3 | 270 | 74 | 69/69 + 181/182 | 270/270 | 269/270 |
| 3878567 | A | CAETHG_3600 → / → CAETHG_3601 | 7 | 7 | - | - | - | - | - |
| 3994749 | G | CAETHG_3707 | 6 | 6 | 261 | 176 | 172/177 + 67/67 | NF | 261/261 |
| 4004460 | G | CAETHG_3715 | 5 | 5 | 113 | 108^ | 108/108 | NF | 113/113 |
| 4025430 | A | CAETHG_3740 | 7 | 7 | 360 | 359* | NF | 360/360 | 356/360 |
| 4030017 | A | CAETHG_3746 ← / → CAETHG_3747 | 7 | 7 | - | - | - | - | - |
| 4044641 | A | CAETHG_3758 | 7 | NF | 174 | 175 | 172/172 | 174/174 | NF |
| 4046087 | G | CAETHG_3761 ← / → CAETHG_3762 | 6 | NF | - | - | - | - | - |
| 4053124 | A | CAETHG_3767 → / → CAETHG_3768 | 13 | NF | - | - | - | - | - |
| 4180142 | T | CAETHG_3902 | 5 | 5 | 359 | 99 | 94/95 + 245/246 | NF | 359/359 |
| 4180905 | G | CAETHG_3903 | 5 | 5 | 359 | 251 | 245/246 + 94/95 | NF | 359/359 |
| 4184490 | A | CAETHG_3905 | 7 | 7 | 767 | 760 | 758/767 | NF | 767/767 |
| 4189892 | T | CAETHG_3911 ← / → CAETHG_3912 | 7 | 7 | - | - | - | - | - |
| 4207332 | T | CAETHG_3925 → / → CAETHG_3926 | 6 | 6 | - | - | - | - | - |
| 4231824 | A | CAETHG_3949 → / → CAETHG_3950 | 8 | NF | - | - | - | - | - |
| 4269911 | C | CAETHG_3986 | 4 | NF | 549 | 170 | 156/156 + 383/383 | 453/454 | NF |
| 4287099 | G | CAETHG_4002 → / → CAETHG_4003 | 6 | NF | - | - | - | - | - |
| 4304216 | T | CAETHG_4019 ← / → CAETHG_4020 | 8 | 8 | - | - | - | - | - |
