# Supplementary material for: Whole genome sequence and manual annotation of Clostridium autoethanogenum, an industrially relevant bacterium
Source: BMC Genomics. 2015 Dec 21;16:1085. doi: 10.1186/s12864-015-2287-5 (PMC4687164; doi:10.1186/s12864-015-2287-5)
Supplement: Additional file 2: — Sanger sequencing of selected discrepancies between the current and Brown et al. sequences. Table showing the region around the discrepancies between our finished genome sequence, confirmed by Sanger sequencing, and the Brown et al. finished genome sequence. (DOCX 23 kb) [file 12864_2015_2287_MOESM2_ESM.docx]

**Additional file 2: Sanger sequencing of selected discrepancies between the current and Brown *et al.* sequences**

| Position | Mutation | Overlapping annotations | Sequence determined by Illumina WGS and confirmed by Sanger sequencing |
| --- | --- | --- | --- |
| 46129 | T | CAETHG_0051 | CRG 1 TAATAGGTGGTATTCTTATGTTTTTTGATTTCCTATTAGT 40 |
|  |  |  | \|\|\|\|\|\|\|\|\|\|\|\|\|\|\|\|\|\|\|\| \|\|\|\|\|\|\|\|\|\|\|\|\|\|\|\|\|\|\| |
|  |  |  | BRO 1 TAATAGGTGGTATTCTTATG-TTTTTGATTTCCTATTAGT 39 |
| 283331 | C | CAETHG_0263 | CRG 1 TTGCTACCCTTTGTTTTTGGCCCCCGCTTAACTGAAATGG 40 |
|  |  |  | \|\|\|\|\|\|\|\|\|\|\|\|\|\|\|\|\|\|\|\| \|\|\|\|\|\|\|\|\|\|\|\|\|\|\|\|\|\|\| |
|  |  |  | BRO 1 TTGCTACCCTTTGTTTTTGG-CCCCGCTTAACTGAAATGG 39 |
| 627984 | C | CAETHG_0567 | CRG 1 CAGTTACTGAGGCAGAAAGGCCTTTTAAAGATAGAGATAT 40 |
|  |  |  | \|\|\|\|\|\|\|\|\|\|\|\|\|\|\|\|\|\|\|\| \|\|\|\|\|\|\|\|\|\|\|\|\|\|\|\|\|\|\| |
|  |  |  | BRO 1 CAGTTACTGAGGCAGAAAGG-CTTTTAAAGATAGAGATAT 39 |
| 656810 | T | CAETHG_0595 | CRG 1 ATACGAAATCCTCTTGATTATTTTTTCTCTCTTTATCAGA 40 |
|  |  |  | \|\|\|\|\|\|\|\|\|\|\|\|\|\|\|\|\|\|\|\| \|\|\|\|\|\|\|\|\|\|\|\|\|\|\|\|\|\|\| |
|  |  |  | BRO 1 ATACGAAATCCTCTTGATTA-TTTTTCTCTCTTTATCAGA 39 |
| 928129 | C | CAETHG_0862 | CRG 1 CAATCTGTTTTAGTTACAGGCCCCCTGCATCCAAGTTTAA 40 |
|  |  |  | \|\|\|\|\|\|\|\|\|\|\|\|\|\|\|\|\|\|\|\| \|\|\|\|\|\|\|\|\|\|\|\|\|\|\|\|\|\|\| |
|  |  |  | BRO 1 CAATCTGTTTTAGTTACAGG-CCCCTGCATCCAAGTTTAA 39 |
| 985484 | C | CAETHG_0915 | CRG 1 TCACTCTTATCTATAGTAAGCCCCAGCGACCAATTTAAGT 40 |
|  |  |  | \|\|\|\|\|\|\|\|\|\|\|\|\|\|\|\|\|\|\|\| \|\|\|\|\|\|\|\|\|\|\|\|\|\|\|\|\|\|\| |
|  |  |  | BRO 1 TCACTCTTATCTATAGTAAG-CCCAGCGACCAATTTAAGT 39 |
| 1106176 | A | CAETHG_1030 | CRG 1 TCCACAAAGCAGTGCTGGACAAAAAACCCACCTTTCTAAC 40 |
|  |  |  | \|\|\|\|\|\|\|\|\|\|\|\|\|\|\|\|\|\|\|\| \|\|\|\|\|\|\|\|\|\|\|\|\|\|\|\|\|\|\| |
|  |  |  | BRO 1 TCCACAAAGCAGTGCTGGAC-AAAAACCCACCTTTCTAAC 39 |
| 1457002 | C | CAETHG_1363 | CRG 1 TTTAATACCCTCGCAGGCATCCCCCCTACAATCAACCTCT 40 |
|  |  |  | \|\|\|\|\|\|\|\|\|\|\|\|\|\|\|\|\|\|\|\| \|\|\|\|\|\|\|\|\|\|\|\|\|\|\|\|\|\|\| |
|  |  |  | BRO 1 TTTAATACCCTCGCAGGCAT-CCCCCTACAATCAACCTCT 39 |
| 1603900 | T | CAETHG_1501 | CRG 1 TAATTTCCTCGTCATCTATATTTTTTTTCTTTATATAAGA 40 |
|  |  |  | \|\|\|\|\|\|\|\|\|\|\|\|\|\|\|\|\|\|\|\| \|\|\|\|\|\|\|\|\|\|\|\|\|\|\|\|\|\|\| |
|  |  |  | BRO 1 TAATTTCCTCGTCATCTATA-TTTTTTTCTTTATATAAGA 39 |
| 1620246 | T | CAETHG_1521 | CRG 1 TTATTATTTTGCATTTCTAATTTTTTATATGTTTTAAGCT 40 |
|  |  |  | \|\|\|\|\|\|\|\|\|\|\|\|\|\|\|\|\|\|\|\| \|\|\|\|\|\|\|\|\|\|\|\|\|\|\|\|\|\|\| |
|  |  |  | BRO 1 TTATTATTTTGCATTTCTAA-TTTTTATATGTTTTAAGCT 39 |
| 2222019 | T | CAETHG_2078 | CRG 1 TAGGACTTAAGCCAATTATATTTTTTTTAAACACCTTACA 40 |
|  |  |  | \|\|\|\|\|\|\|\|\|\|\|\|\|\|\|\|\|\|\|\| \|\|\|\|\|\|\|\|\|\|\|\|\|\|\|\|\|\|\| |
|  |  |  | BRO 1 TAGGACTTAAGCCAATTATA-TTTTTTTAAACACCTTACA 39 |
| 2352969 | T | CAETHG_2212, CAETHG_2213 | CRG 1 TAATAGATACTATTTGTCAGTTGTTTAATAATATAGACAG 40 |
|  |  |  | \|\|\|\|\|\|\|\|\|\|\|\|\|\|\|\|\|\|\|\| \|\|\|\|\|\|\|\|\|\|\|\|\|\|\|\|\|\|\| |
|  |  |  | BRO 1 TAATAGATACTATTTGTCAG-TGTTTAATAATATAGACAG 39 |
| 2596835 | G | CAETHG_2429 | CRG 1 AACATTACCTCTTGTAAGTAGGGGGGGAAGTTCTATGCTA 40 |
|  |  |  | \|\|\|\|\|\|\|\|\|\|\|\|\|\|\|\|\|\|\|\| \|\|\|\|\|\|\|\|\|\|\|\|\|\|\|\|\|\|\| |
|  |  |  | BRO 1 AACATTACCTCTTGTAAGTA-GGGGGGAAGTTCTATGCTA 39 |
| 2683087 | C | CAETHG_2503 | CRG 1 ATTTTTCATTTTGTTCCTTGCCCCTTACTTTTGATTTAGT 40 |
|  |  |  | \|\|\|\|\|\|\|\|\|\|\|\|\|\|\|\|\|\|\|\| \|\|\|\|\|\|\|\|\|\|\|\|\|\|\|\|\|\|\| |
|  |  |  | BRO 1 ATTTTTCATTTTGTTCCTTG-CCCTTACTTTTGATTTAGT 39 |
| 2805023 | A | CAETHG_2601, CAETHG_2602 | CRG 1 TTTATTACAGATTTATCTATAAAAAAAGCACATAAGATAA 40 |
|  |  |  | \|\|\|\|\|\|\|\|\|\|\|\|\|\|\|\|\|\|\|\| \|\|\|\|\|\|\|\|\|\|\|\|\|\|\|\|\|\|\| |
|  |  |  | BRO 1 TTTATTACAGATTTATCTAT-AAAAAAGCACATAAGATAA 39 |
| 2852812 | T | CAETHG_2647 | CRG 1 TTATACATGAAATAGAATTATTTTTTTTAATAACTATTAT 40 |
|  |  |  | \|\|\|\|\|\|\|\|\|\|\|\|\|\|\|\|\|\|\|\| \|\|\|\|\|\|\|\|\|\|\|\|\|\|\|\|\|\|\| |
|  |  |  | BRO 1 TTATACATGAAATAGAATTA-TTTTTTTAATAACTATTAT 39 |
| 3076804 | A | CAETHG_2840 | CRG 1 ATTAAAAATAAAGTATTTGGAAAAAAAATTAAGCAAAAAA 40 |
|  |  |  | \|\|\|\|\|\|\|\|\|\|\|\|\|\|\|\|\|\|\|\| \|\|\|\|\|\|\|\|\|\|\|\|\|\|\|\|\|\|\| |
|  |  |  | BRO 1 ATTAAAAATAAAGTATTTGG-AAAAAAATTAAGCAAAAAA 39 |
| 3396986 | G | CAETHG_3132, CAETHG_3133 | CRG 1 GAGAAAACTGGTATTGGTAAGGGGGAAGTTCTATTAATAA 40 |
|  |  |  | \|\|\|\|\|\|\|\|\|\|\|\|\|\|\|\|\|\|\|\| \|\|\|\|\|\|\|\|\|\|\|\|\|\|\|\|\|\|\| |
|  |  |  | BRO 1 GAGAAAACTGGTATTGGTAA-GGGGAAGTTCTATTAATAA 39 |
| 3468796 | G | CAETHG_3212 | CRG 1 ATGGCAATTAGCTAAAAAGAGGGGGAGTAAATAGTATGGA 40 |
|  |  |  | \|\|\|\|\|\|\|\|\|\|\|\|\|\|\|\|\|\|\|\| \|\|\|\|\|\|\|\|\|\|\|\|\|\|\|\|\|\|\| |
|  |  |  | BRO 1 ATGGCAATTAGCTAAAAAGA-GGGGAGTAAATAGTATGGA 39 |
| 3752592 | G | CAETHG_3500 | CRG 1 ACTAGTATTGCCGCGCTTTTGGGGGACTGAAGGTGTATGG 40 |
|  |  |  | \|\|\|\|\|\|\|\|\|\|\|\|\|\|\|\|\|\|\|\| \|\|\|\|\|\|\|\|\|\|\|\|\|\|\|\|\|\|\| |
|  |  |  | BRO 1 ACTAGTATTGCCGCGCTTTT-GGGGACTGAAGGTGTATGG 39 |
| 3786709 | T | CAETHG_3531 | CRG 1 AAGGAAATTAAAGATAAAGATTTTTTGAAAAAATTAGTAG 40 |
|  |  |  | \|\|\|\|\|\|\|\|\|\|\|\|\|\|\|\|\|\|\|\| \|\|\|\|\|\|\|\|\|\|\|\|\|\|\|\|\|\|\| |
|  |  |  | BRO 1 AAGGAAATTAAAGATAAAGA-TTTTTGAAAAAATTAGTAG 39 |
| 3877937 | A | CAETHG_3599 | CRG 1 TTTATGAAAAATAATAATATAAATGTATATGATTTTGTAA 40 |
|  |  |  | \|\|\|\|\|\|\|\|\|\|\|\|\|\|\|\|\|\|\|\| \|\|\|\|\|\|\|\|\|\|\|\|\|\|\|\|\|\|\| |
|  |  |  | BRO 1 TTTATGAAAAATAATAATAT-AATGTATATGATTTTGTAA 39 |
| 3994749 | G | CAETHG_3707 | CRG 1 GAAAAGATAGTAAAAGATGCGGGGGGATTTATATATTGTG 40 |
|  |  |  | \|\|\|\|\|\|\|\|\|\|\|\|\|\|\|\|\|\|\|\| \|\|\|\|\|\|\|\|\|\|\|\|\|\|\|\|\|\|\| |
|  |  |  | BRO 1 GAAAAGATAGTAAAAGATGC-GGGGGATTTATATATTGTG 39 |
| 4180142 | T | CAETHG_3902 | CRG 1 TCCCAAACCACGTCTAGTTGTTTTTGGTGGTGGACACATA 40 |
|  |  |  | \|\|\|\|\|\|\|\|\|\|\|\|\|\|\|\|\|\|\|\| \|\|\|\|\|\|\|\|\|\|\|\|\|\|\|\|\|\|\| |
|  |  |  | BRO 1 TCCCAAACCACGTCTAGTTG-TTTTGGTGGTGGACACATA 39 |
| 3468964 | C → A | CAETHG_3212 | CRG 1 ATTAATCGAAGAGAAAAAGCAAGACTTAGTAGTACTGGAT 40 |
|  |  |  | \|\|\|\|\|\|\|\|\|\|\|\|\|\|\|\|\|\|\|\|.\|\|\|\|\|\|\|\|\|\|\|\|\|\|\|\|\|\|\| |
|  |  |  | BRO 1 ATTAATCGAAGAGAAAAAGCCAGACTTAGTAGTACTGGAT 40 |
