# Supplementary material for: Whole genome sequence and manual annotation of Clostridium autoethanogenum, an industrially relevant bacterium
Source: BMC Genomics. 2015 Dec 21;16:1085. doi: 10.1186/s12864-015-2287-5 (PMC4687164; doi:10.1186/s12864-015-2287-5)

|  | Count | Percentage of reads | Average length | Number of bases | Percentage of bases |
| --- | --- | --- | --- | --- | --- |
| References | 1 | - | 4,352,205.00 | 4,352,205 | - |
| Mapped reads | 3,508,407 | 97.51% | 249.91 | 876,795,225 | 97.51% |
| Not mapped reads | 89,517 | 2.49% | 250.06 | 22,384,530 | 2.49% |
| Reads in pairs | 3,389,806 | 94.22% | 543.31 | 849,038,084 | 94.42% |
| Broken paired reads | 118,601 | 3.30% | 234.04 | 27,757,141 | 3.09% |
| Total reads | 3,597,924 | 100.00% | 249.92 | 899,179,755 | 100.00% |

**1 Mapping summary report**

- 1. **Summary statistics**

**1.2 Distribution of read length**


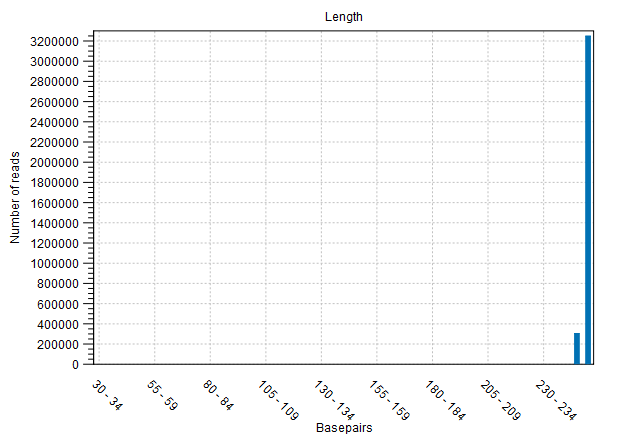


**1.3 Distribution of mapped read length**


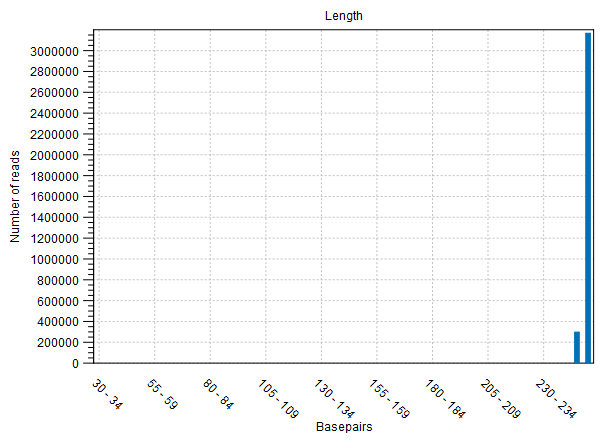


**1.4 Distribution of un-mapped read length**


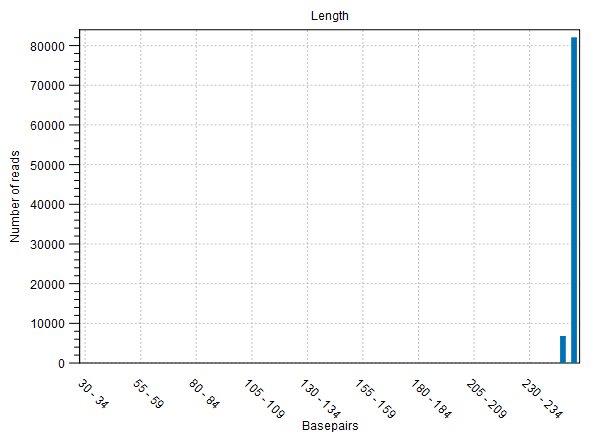


**1.5 Paired reads distance distribution**


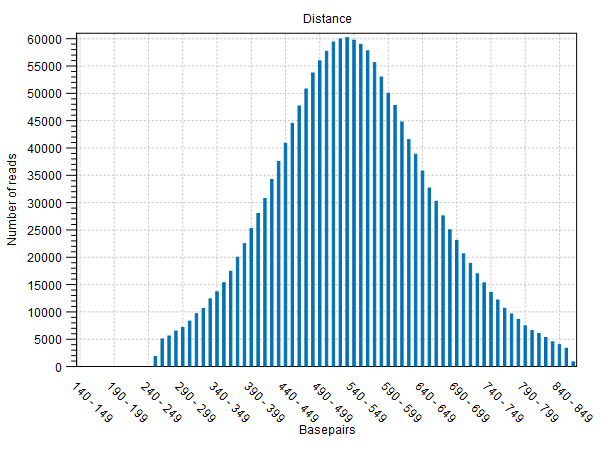

Supplement: Additional file 4: — Illumina mapping summary report. (DOCX 84 kb) [file 12864_2015_2287_MOESM4_ESM.docx]
