# Supplementary material for: Whole genome sequence and manual annotation of Clostridium autoethanogenum, an industrially relevant bacterium
Source: BMC Genomics. 2015 Dec 21;16:1085. doi: 10.1186/s12864-015-2287-5 (PMC4687164; doi:10.1186/s12864-015-2287-5)
Supplement: Additional file 5: — List of primers used in this study. A list of a forward and reverse primers used in this study for verification of whole genome sequencing. (DOCX 17 kb) [file 12864_2015_2287_MOESM5_ESM.docx]

**Additional file 4: List of primers used in this study**

| Position | Mutation | Overlapping annotations | Primer | Forward primer | Primer | Reverse primer |
| --- | --- | --- | --- | --- | --- | --- |
| 46129 | T | CAETHG_0051 | 0051F | GCTAATACACCTAAAAATAGAATTTC | 0051R | CTGATGCTCCTTTTGTTG |
| 283331 | C | CAETHG_0263 | 0263F | CATATACAAATGTTATCCCCAG | 0263R | GAATGTATTTGATAATGTAGCCTATG |
| 627984 | C | CAETHG_0567 | 0567F | GAATATGGGAAAAAGTTGATAGTC | 0567R | CCTTATCTGTAACAGCGTTATC |
| 656810 | T | CAETHG_0595 | 0595F | GGAGCAAACATTAAAATGC | 0595R | CTAATGAAGAACAATCTGGC |
| 928129 | C | CAETHG_0862 | 0862F | CTCCTCACTAATACCTTACAAATG | 0862R | GGAATTACCATTCATGATAGTTG |
| 985484 | C | CAETHG_0915 | 0915F | GATAATTCCAGGGCATAC | 0915R | GCTAAATAAAGATAATACTATAGAAGATG |
| 1106176 | A | CAETHG_1030 | 1030F | CAATAACACCAACATACCATAC | 1030R | GCACTTTTTATTATGAATTATTTGC |
| 1457002 | C | CAETHG_1363 | 1363F | GACAACCATACCTGTAGGTTC | 1363R | GAAATTCGCCAGTAGACATATAC |
| 1603900 | T | CAETHG_1501 | 1501F | CTATATTTTCTACATTGGAACATACC | 1501R | CGAGAAGTGATTTTTGAGG |
| 1620246 | T | CAETHG_1521 | 1521F | GTTTACATTTCACCATACATG | 1521R | CTTTAGGATATGGGCGTAAG |
| 2222019 | T | CAETHG_2078 | 2078F | CTTCATTTTCTCCTATAAATTCATC | 2078R | GCTTAAAAAGATGGTAGAGG |
| 2352969 | T | CAETHG_2212, CAETHG_2213 | 2212F | GTGATGCAAATACAGGACC | 2212R | GGAAAAGTACAGACAGGAAG |
| 2596835 | G | CAETHG_2429 | 2429F | CAGATGTATGAGAGCTGCTG | 2429R | CTCTATCTTAGGTCCCACTATC |
| 2683087 | C | CAETHG_2503 | 2503F | GATTTCTCTACTAATTGTTTGTGG | 2503R | CTACGTTATAAGAAATGGAGG |
| 2805023 | A | CAETHG_2601, CAETHG_2602 | 2601F | GAAGACTTAATAATTGTTGAAGG | 2601R | CTGCCACAACTAATCTATAATC |
| 2852812 | T | CAETHG_2647 | 2647F | GCATCAAAATTAGTTAAAAAGAGTAC | 2647R | GAGCCTTGTCCTCATAAG |
| 3076804 | A | CAETHG_2840 | 2840F | CTGTATATCTTCATAAAGATTTTTTG | 2840R | CTAATATCATTCTGCATATCATTG |
| 3396986 | G | CAETHG_3132, CAETHG_3133 | 3132F | GAAGAAATCAAAGACAATAATG | 3132R | CTTATCCTCAATTTGACTTTTAC |
| 3468796 | G | CAETHG_3212 | 3212F | GATACTAAAGATAGCATTTAATATGC | 3212R | CTACTAAGTCTTGCTTTTTCTCTTC |
| 3752592 | G | CAETHG_3500 | 3500F | CCTTATGTTTCCTGTAGCG | 3500R | CATACAATTTTCATTTATATGTTTC |
| 3786709 | T | CAETHG_3531 | 3531F | CGAAGAAATATATTCTCTTACAGC | 3531R | CATGTATTATAAAGTGCTCTTCC |
| 3877937 | A | CAETHG_3599 | 3599F | GTTATAGGAACAGCTTTGAGC | 3599R | GCTATAGGTGCTATAATAACTGTTG |
| 3994749 | G | CAETHG_3707 | 3707F | CTCCATATTCAAGTATGGTATGG | 3707R | GCTGAGGATATTCCAAATCC |
| 4180142 | T | CAETHG_3902 | 3902F | GTACAGAAGAAGGTTCTATTATAAAG | 3902R | CTGCTTCAGGAAATCTTG |
| 3468964 | C → A | CAETHG_3212 | 3212_SNP_F | TTGTCGAATTTATTATTTGTTAAGA | 3212_SNP_R | TTAGCTTACCTTATTTTTTAATCTT |
| 1 | -A | - | SNP8F1 | GAGAAGATCAGGACAACAGTG | SNP8R1 | CGGATAAAATATCATGTCC |
